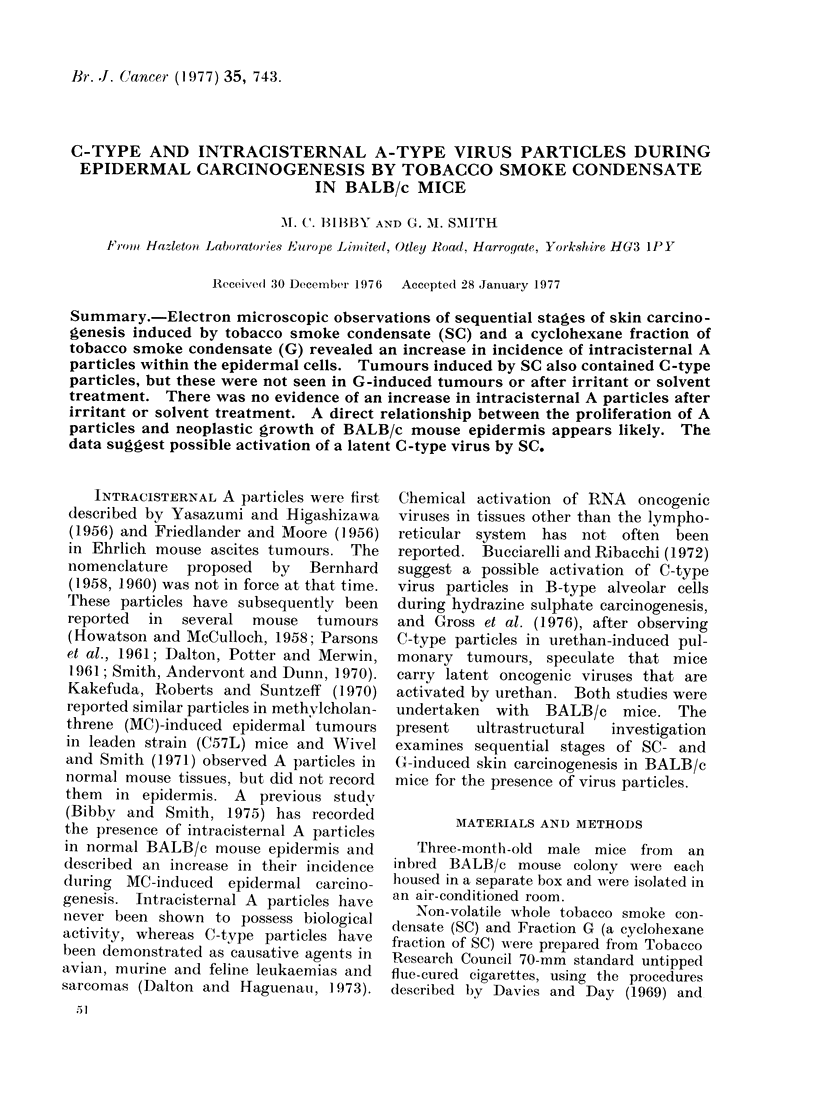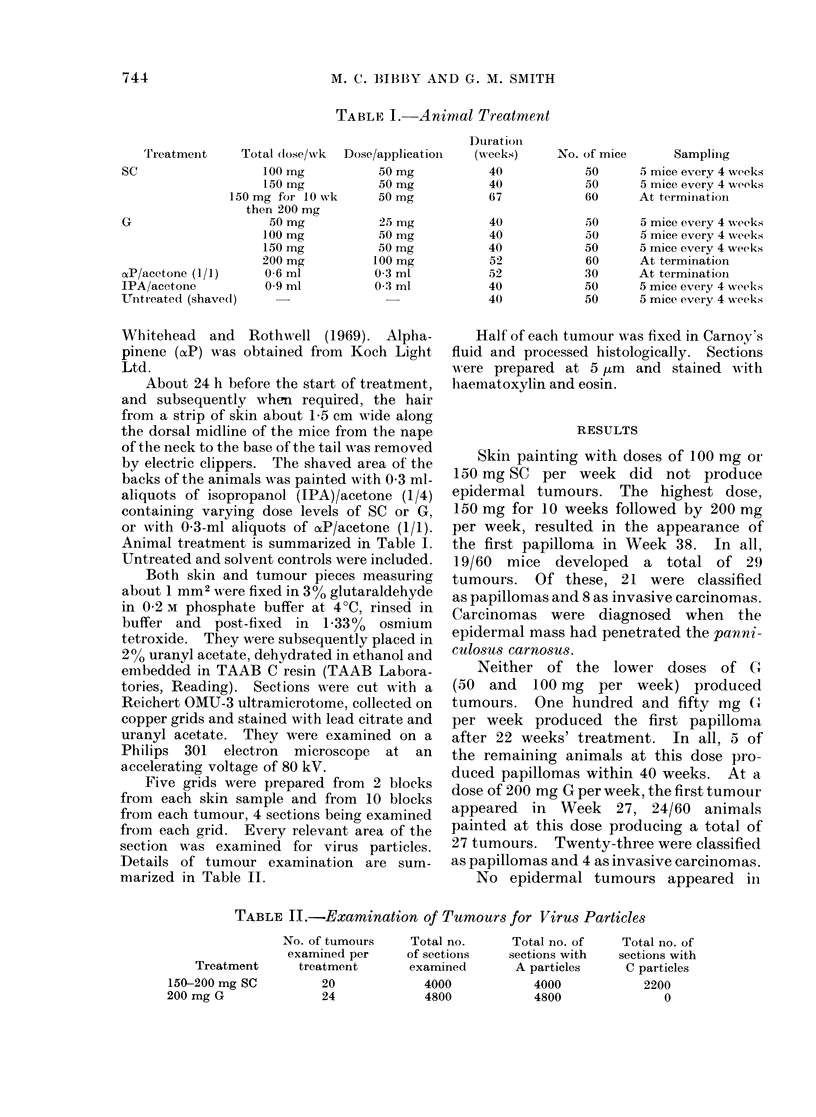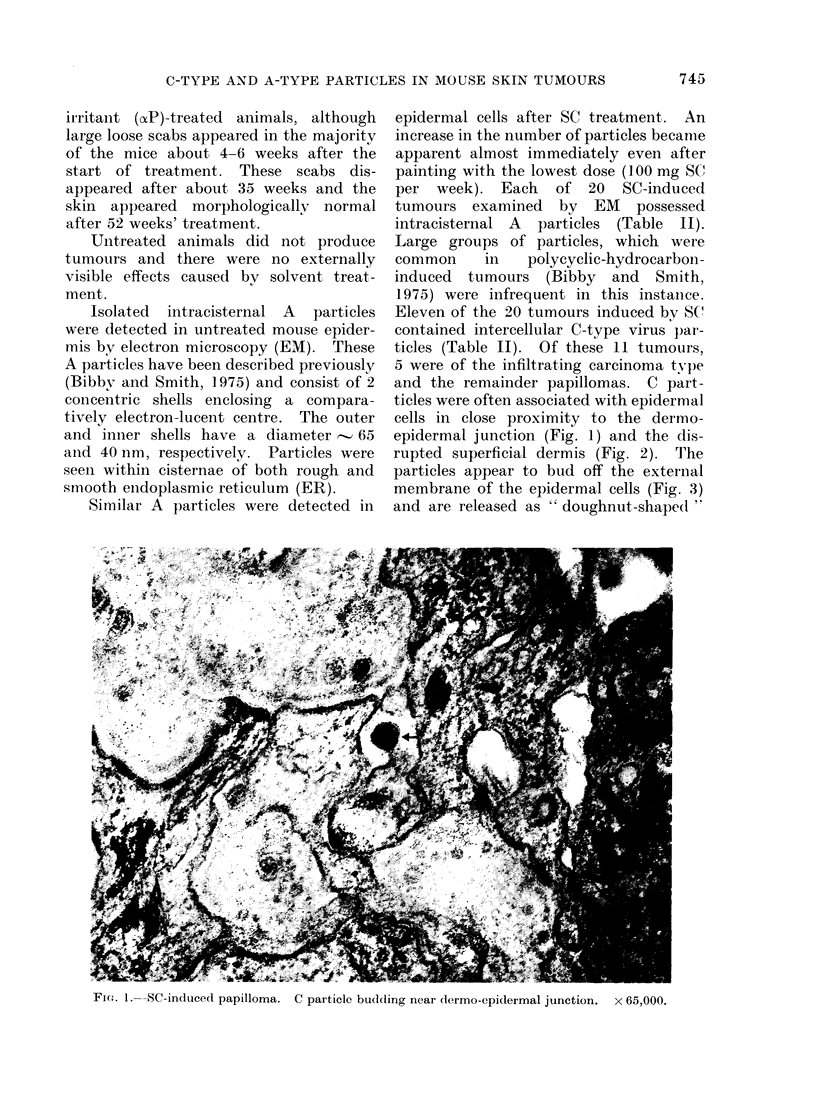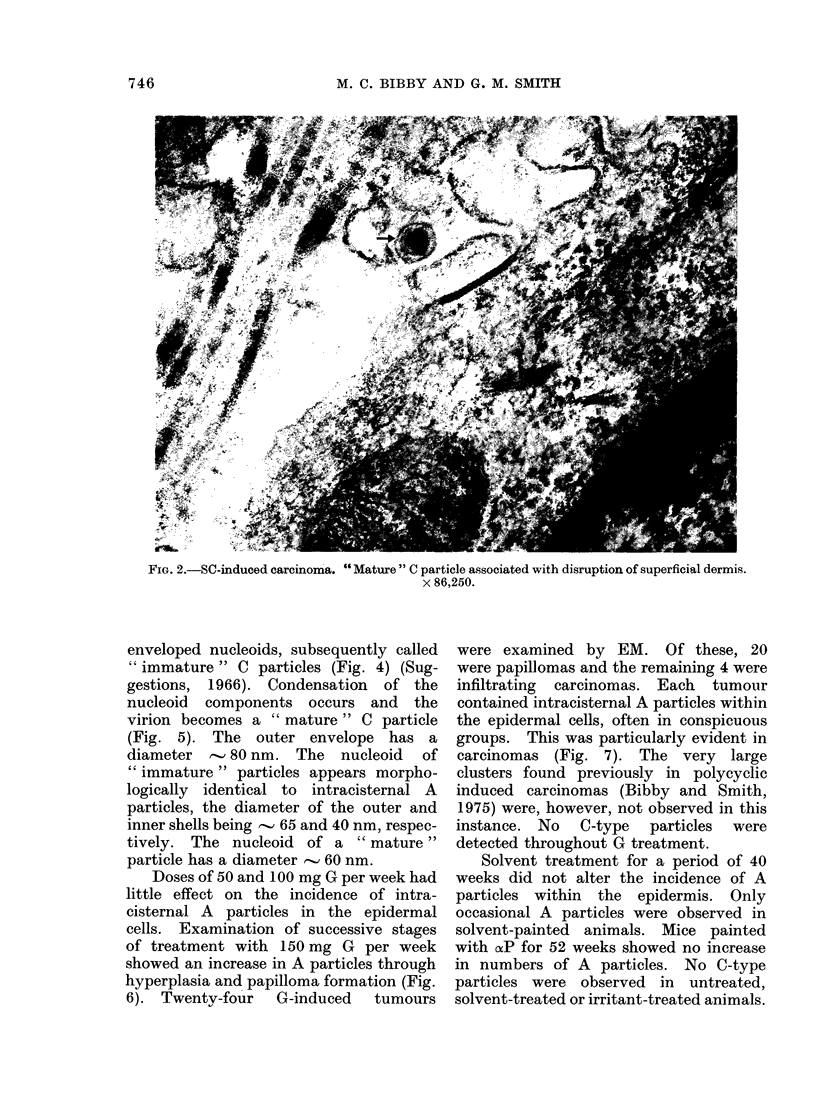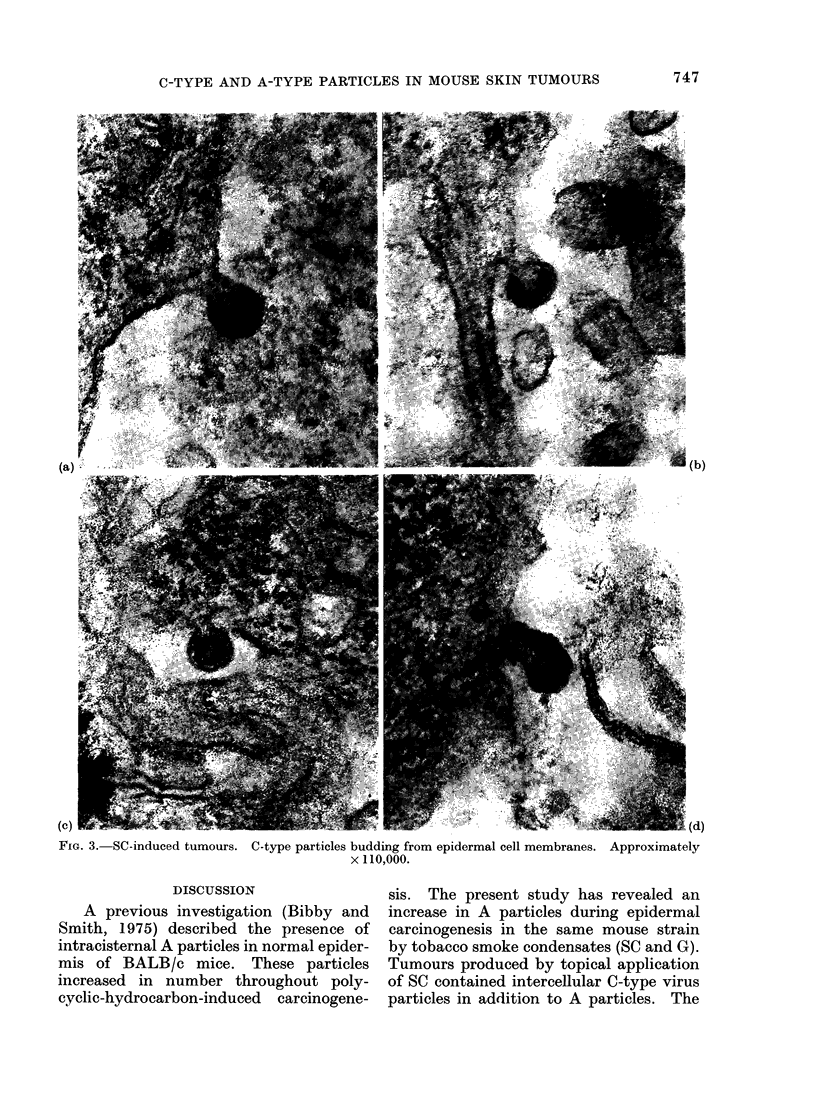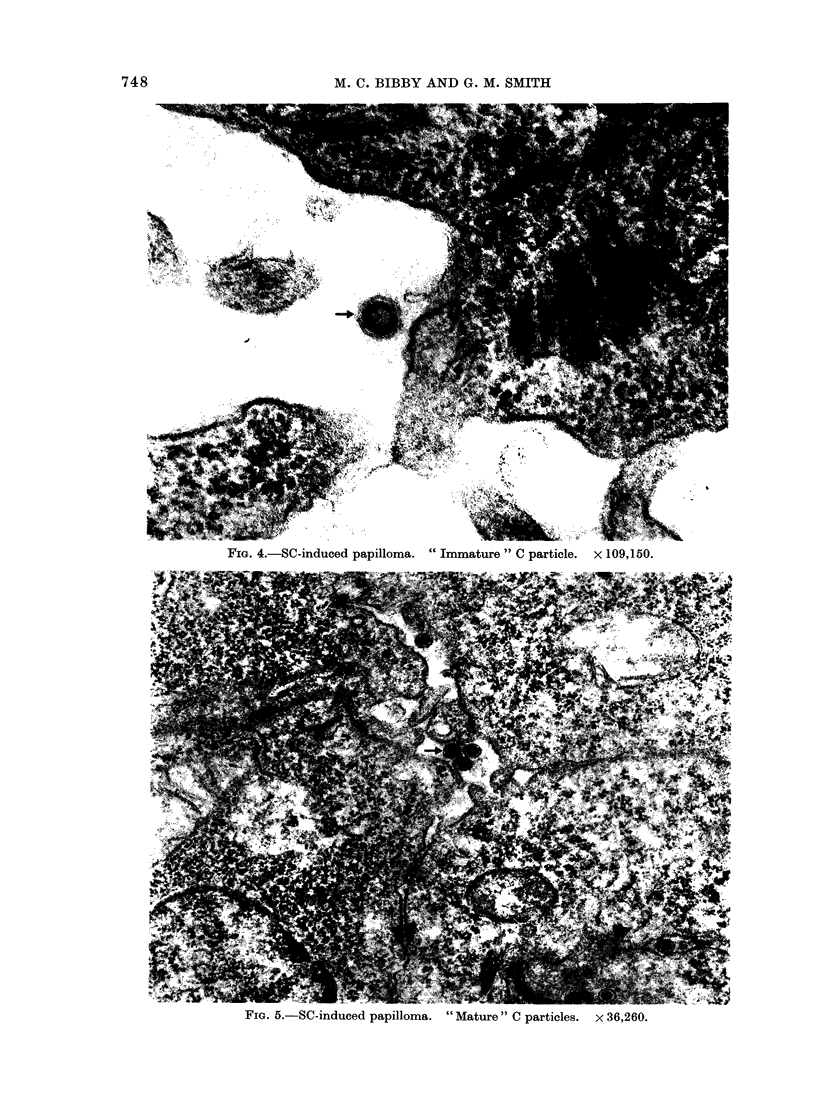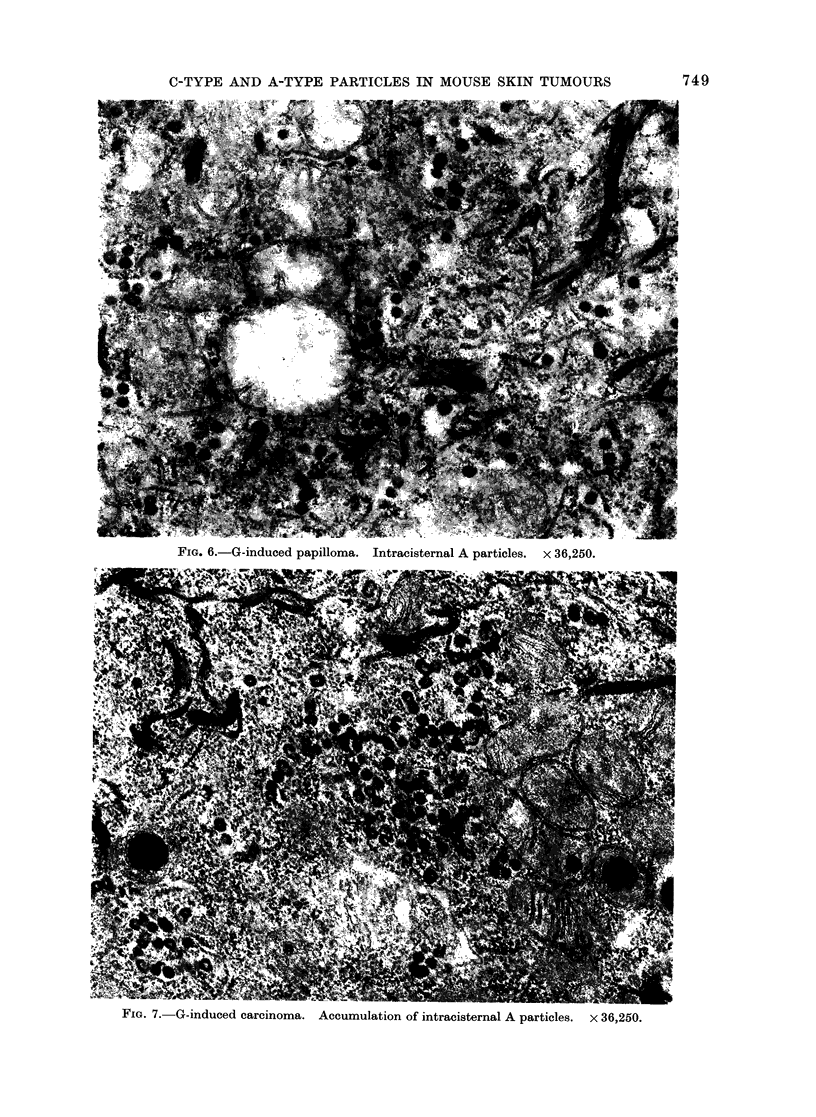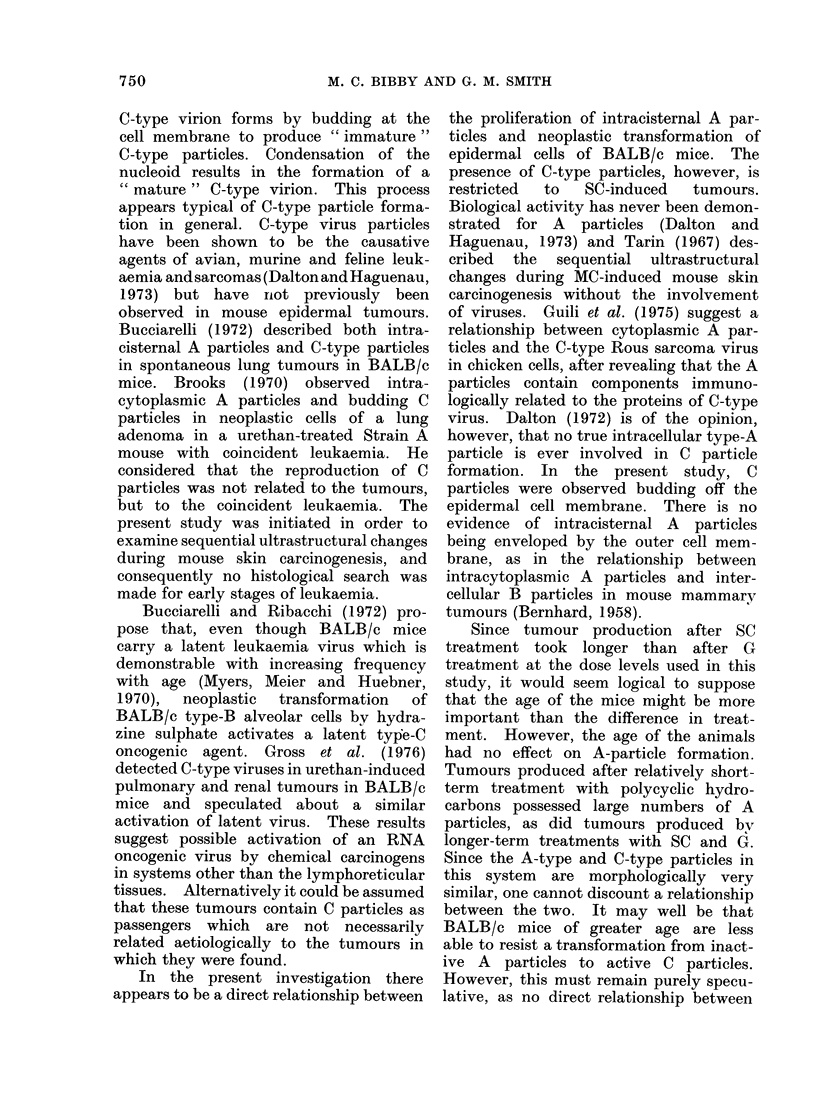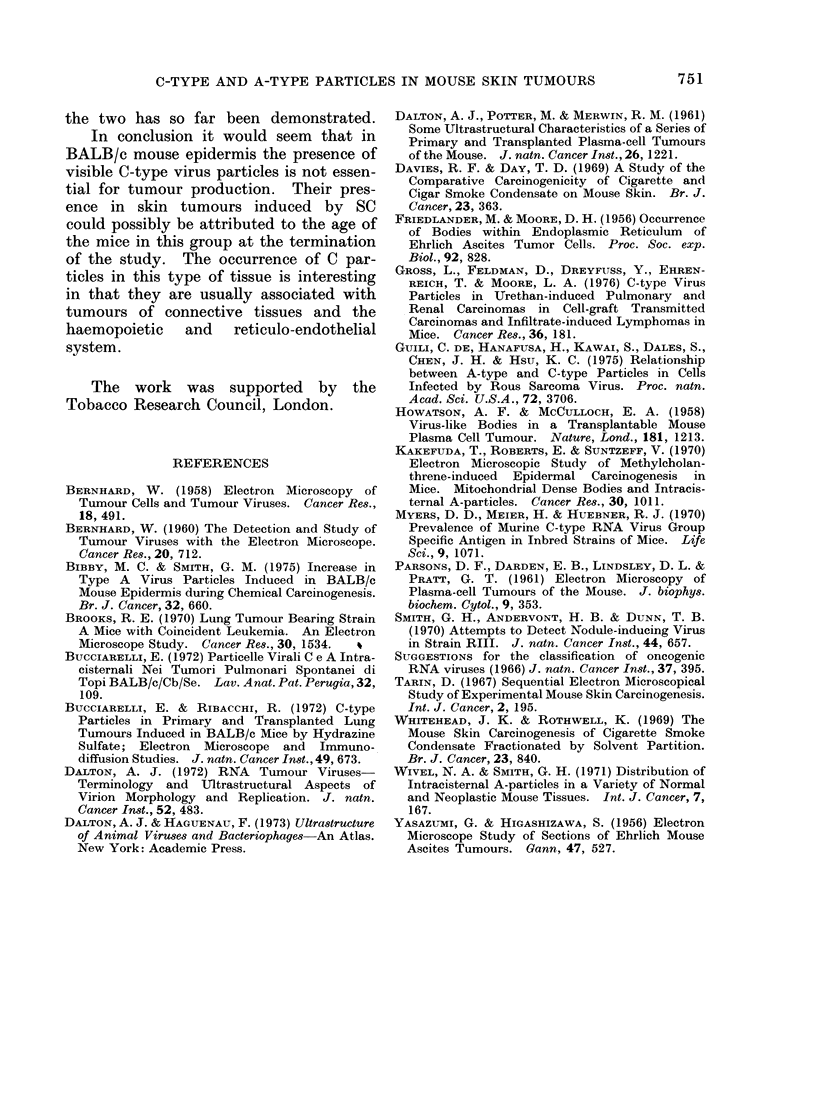# C-type and intracisternal A-type virus particles during epidermal carcinogenesis by tobacco smoke condensate in BALB/c mice.

**DOI:** 10.1038/bjc.1977.115

**Published:** 1977-06

**Authors:** M. C. Bibby, G. M. Smith

## Abstract

**Images:**


*Br. J. Cancer* (1977) **35**, 743.

### C-TYPE AND INTRACISTERNAL A-TYPE VIRUS PARTICLES DURING EPIDERMAL CARCINOGENESIS BY TOBACCO SMOKE CONDENSATE IN BALB/c MICE

M. C. BIBBY AND G. M. SMITH

*From Hazleton Laboratories Europe Limited, Otley Road, Harrogate, Yorkshire HG3 1PY*

Received 30 December 1976 Accepted 28 January 1977

**Summary.—Electron microscopic observations of sequential stages of skin carcinogenesis induced by tobacco smoke condensate (SC) and a cyclohexane fraction of tobacco smoke condensate (G) revealed an increase in incidence of intracisternal A particles within the epidermal cells. Tumours induced by SC also contained C-type particles, but these were not seen in G-induced tumours or after irritant or solvent treatment. There was no evidence of an increase in intracisternal A particles after irritant or solvent treatment. A direct relationship between the proliferation of A particles and neoplastic growth of BALB/c mouse epidermis appears likely. The data suggest possible activation of a latent C-type virus by SC.**

INTRACISTERNAL A particles were first described by Yasazumi and Higashizawa (1956) and Friedlander and Moore (1956) in Ehrlich mouse ascites tumours. The nomenclature proposed by Bernhard (1958, 1960) was not in force at that time. These particles have subsequently been reported in several mouse tumours (Howatson and McCulloch, 1958; Parsons *et al.*, 1961; Dalton, Potter and Merwin, 1961; Smith, Andervont and Dunn, 1970). Kakefuda, Roberts and Suntzeff (1970) reported similar particles in methylcholanthrene (MC)-induced epidermal tumours in leaden strain (C57L) mice and Wivel and Smith (1971) observed A particles in normal mouse tissues, but did not record them in epidermis. A previous study (Bibby and Smith, 1975) has recorded the presence of intracisternal A particles in normal BALB/c mouse epidermis and described an increase in their incidence during MC-induced epidermal carcinogenesis. Intracisternal A particles have never been shown to possess biological activity, whereas C-type particles have been demonstrated as causative agents in avian, murine and feline leukaemias and sarcomas (Dalton and Haguenau, 1973). Chemical activation of RNA oncogenic viruses in tissues other than the lymphoreticular system has not often been reported. Bucciarelli and Ribacchi (1972) suggest a possible activation of C-type virus particles in B-type alveolar cells during hydrazine sulphate carcinogenesis, and Gross *et al.* (1976), after observing C-type particles in urethan-induced pulmonary tumours, speculate that mice carry latent oncogenic viruses that are activated by urethan. Both studies were undertaken with BALB/c mice. The present ultrastructural investigation examines sequential stages of SC- and G-induced skin carcinogenesis in BALB/c mice for the presence of virus particles.

### MATERIALS AND METHODS

Three-month-old male mice from an inbred BALB/c mouse colony were each housed in a separate box and were isolated in an air-conditioned room.

Non-volatile whole tobacco smoke condensate (SC) and Fraction G (a cyclohexane fraction of SC) were prepared from Tobacco Research Council 70-mm standard untipped flue-cured cigarettes, using the procedures described by Davies and Day (1969) and

TABLE I.—*Animal Treatment*

| Treatment | Total dose/wk | Dose/application | Duration (weeks) | No. of mice | Sampling |
|---|---|---|---|---|---|
| SC | 100 mg | 50 mg | 40 | 50 | 5 mice every 4 weeks |
| | 150 mg | 50 mg | 40 | 50 | 5 mice every 4 weeks |
| | 150 mg for 10 wk then 200 mg | 50 mg | 67 | 60 | At termination |
| G | 50 mg | 25 mg | 40 | 50 | 5 mice every 4 weeks |
| | 100 mg | 50 mg | 40 | 50 | 5 mice every 4 weeks |
| | 150 mg | 50 mg | 40 | 50 | 5 mice every 4 weeks |
| | 200 mg | 100 mg | 52 | 60 | At termination |
| αP/acetone (1/1) | 0·6 ml | 0·3 ml | 52 | 30 | At termination |
| IPA/acetone | 0·9 ml | 0·3 ml | 40 | 50 | 5 mice every 4 weeks |
| Untreated (shaved) | — | — | 40 | 50 | 5 mice every 4 weeks |

Whitehead and Rothwell (1969). Alpha-pinene (αP) was obtained from Koch Light Ltd.

About 24 h before the start of treatment, and subsequently when required, the hair from a strip of skin about 1·5 cm wide along the dorsal midline of the mice from the nape of the neck to the base of the tail was removed by electric clippers. The shaved area of the backs of the animals was painted with 0·3 ml-aliquots of isopropanol (IPA)/acetone (1/4) containing varying dose levels of SC or G, or with 0·3-ml aliquots of αP/acetone (1/1). Animal treatment is summarized in Table I. Untreated and solvent controls were included.

Both skin and tumour pieces measuring about 1 $mm^2$ were fixed in 3% glutaraldehyde in 0·2 M phosphate buffer at 4°C, rinsed in buffer and post-fixed in 1·33% osmium tetroxide. They were subsequently placed in 2% uranyl acetate, dehydrated in ethanol and embedded in TAAB C resin (TAAB Laboratories, Reading). Sections were cut with a Reichert OMU-3 ultramicrotome, collected on copper grids and stained with lead citrate and uranyl acetate. They were examined on a Philips 301 electron microscope at an accelerating voltage of 80 kV.

Five grids were prepared from 2 blocks from each skin sample and from 10 blocks from each tumour, 4 sections being examined from each grid. Every relevant area of the section was examined for virus particles. Details of tumour examination are summarized in Table II.

Half of each tumour was fixed in Carnoy's fluid and processed histologically. Sections were prepared at 5 μm and stained with haematoxylin and eosin.

### RESULTS

Skin painting with doses of 100 mg or 150 mg SC per week did not produce epidermal tumours. The highest dose, 150 mg for 10 weeks followed by 200 mg per week, resulted in the appearance of the first papilloma in Week 38. In all, 19/60 mice developed a total of 29 tumours. Of these, 21 were classified as papillomas and 8 as invasive carcinomas. Carcinomas were diagnosed when the epidermal mass had penetrated the *panniculosus carnosus*.

Neither of the lower doses of G (50 and 100 mg per week) produced tumours. One hundred and fifty mg G per week produced the first papilloma after 22 weeks' treatment. In all, 5 of the remaining animals at this dose produced papillomas within 40 weeks. At a dose of 200 mg G per week, the first tumour appeared in Week 27, 24/60 animals painted at this dose producing a total of 27 tumours. Twenty-three were classified as papillomas and 4 as invasive carcinomas.

No epidermal tumours appeared in

TABLE II.—*Examination of Tumours for Virus Particles*

| Treatment | No. of tumours examined per treatment | Total no. of sections examined | Total no. of sections with A particles | Total no. of sections with C particles |
|---|---|---|---|---|
| 150–200 mg SC | 20 | 4000 | 4000 | 2200 |
| 200 mg G | 24 | 4800 | 4800 | 0 |

irritant (αP)-treated animals, although large loose scabs appeared in the majority of the mice about 4–6 weeks after the start of treatment. These scabs disappeared after about 35 weeks and the skin appeared morphologically normal after 52 weeks' treatment.

Untreated animals did not produce tumours and there were no externally visible effects caused by solvent treatment.

Isolated intracisternal A particles were detected in untreated mouse epidermis by electron microscopy (EM). These A particles have been described previously (Bibby and Smith, 1975) and consist of 2 concentric shells enclosing a comparatively electron-lucent centre. The outer and inner shells have a diameter ~ 65 and 40 nm, respectively. Particles were seen within cisternae of both rough and smooth endoplasmic reticulum (ER).

Similar A particles were detected in epidermal cells after SC treatment. An increase in the number of particles became apparent almost immediately even after painting with the lowest dose (100 mg SC per week). Each of 20 SC-induced tumours examined by EM possessed intracisternal A particles (Table II). Large groups of particles, which were common in polycyclic-hydrocarbon-induced tumours (Bibby and Smith, 1975) were infrequent in this instance. Eleven of the 20 tumours induced by SC contained intercellular C-type virus particles (Table II). Of these 11 tumours, 5 were of the infiltrating carcinoma type and the remainder papillomas. C particles were often associated with epidermal cells in close proximity to the dermo-epidermal junction (Fig. 1) and the disrupted superficial dermis (Fig. 2). The particles appear to bud off the external membrane of the epidermal cells (Fig. 3) and are released as "doughnut-shaped"

FIG. 1.—SC-induced papilloma. C particle budding near dermo-epidermal junction. ×65,000.

FIG. 2.—SC-induced carcinoma. "Mature" C particle associated with disruption of superficial dermis. × 86,250.

enveloped nucleoids, subsequently called "immature" C particles (Fig. 4) (Suggestions, 1966). Condensation of the nucleoid components occurs and the virion becomes a "mature" C particle (Fig. 5). The outer envelope has a diameter ~ 80 nm. The nucleoid of "immature" particles appears morphologically identical to intracisternal A particles, the diameter of the outer and inner shells being ~ 65 and 40 nm, respectively. The nucleoid of a "mature" particle has a diameter ~ 60 nm.

Doses of 50 and 100 mg G per week had little effect on the incidence of intracisternal A particles in the epidermal cells. Examination of successive stages of treatment with 150 mg G per week showed an increase in A particles through hyperplasia and papilloma formation (Fig. 6). Twenty-four G-induced tumours were examined by EM. Of these, 20 were papillomas and the remaining 4 were infiltrating carcinomas. Each tumour contained intracisternal A particles within the epidermal cells, often in conspicuous groups. This was particularly evident in carcinomas (Fig. 7). The very large clusters found previously in polycyclic induced carcinomas (Bibby and Smith, 1975) were, however, not observed in this instance. No C-type particles were detected throughout G treatment.

Solvent treatment for a period of 40 weeks did not alter the incidence of A particles within the epidermis. Only occasional A particles were observed in solvent-painted animals. Mice painted with αP for 52 weeks showed no increase in numbers of A particles. No C-type particles were observed in untreated, solvent-treated or irritant-treated animals.

FIG. 3.—SC-induced tumours. C-type particles budding from epidermal cell membranes. Approximately ×110,000.

### DISCUSSION

A previous investigation (Bibby and Smith, 1975) described the presence of intracisternal A particles in normal epidermis of BALB/c mice. These particles increased in number throughout polycyclic-hydrocarbon-induced carcinogenesis. The present study has revealed an increase in A particles during epidermal carcinogenesis in the same mouse strain by tobacco smoke condensates (SC and G). Tumours produced by topical application of SC contained intercellular C-type virus particles in addition to A particles. The

FIG. 4.—SC-induced papilloma. " Immature " C particle. ×109,150.

FIG. 5.—SC-induced papilloma. "Mature" C particles. ×36,260.

FIG. 6.—G-induced papilloma. Intracisternal A particles. ×36,250.

FIG. 7.—G-induced carcinoma. Accumulation of intracisternal A particles. ×36,250.

C-type virion forms by budding at the cell membrane to produce " immature " C-type particles. Condensation of the nucleoid results in the formation of a " mature " C-type virion. This process appears typical of C-type particle formation in general. C-type virus particles have been shown to be the causative agents of avian, murine and feline leukaemia and sarcomas (Dalton and Haguenau, 1973) but have not previously been observed in mouse epidermal tumours. Bucciarelli (1972) described both intracisternal A particles and C-type particles in spontaneous lung tumours in BALB/c mice. Brooks (1970) observed intracytoplasmic A particles and budding C particles in neoplastic cells of a lung adenoma in a urethan-treated Strain A mouse with coincident leukaemia. He considered that the reproduction of C particles was not related to the tumours, but to the coincident leukaemia. The present study was initiated in order to examine sequential ultrastructural changes during mouse skin carcinogenesis, and consequently no histological search was made for early stages of leukaemia.

Bucciarelli and Ribacchi (1972) propose that, even though BALB/c mice carry a latent leukaemia virus which is demonstrable with increasing frequency with age (Myers, Meier and Huebner, 1970), neoplastic transformation of BALB/c type-B alveolar cells by hydrazine sulphate activates a latent type-C oncogenic agent. Gross *et al.* (1976) detected C-type viruses in urethan-induced pulmonary and renal tumours in BALB/c mice and speculated about a similar activation of latent virus. These results suggest possible activation of an RNA oncogenic virus by chemical carcinogens in systems other than the lymphoreticular tissues. Alternatively it could be assumed that these tumours contain C particles as passengers which are not necessarily related aetiologically to the tumours in which they were found.

In the present investigation there appears to be a direct relationship between the proliferation of intracisternal A particles and neoplastic transformation of epidermal cells of BALB/c mice. The presence of C-type particles, however, is restricted to SC-induced tumours. Biological activity has never been demonstrated for A particles (Dalton and Haguenau, 1973) and Tarin (1967) described the sequential ultrastructural changes during MC-induced mouse skin carcinogenesis without the involvement of viruses. Guili *et al.* (1975) suggest a relationship between cytoplasmic A particles and the C-type Rous sarcoma virus in chicken cells, after revealing that the A particles contain components immunologically related to the proteins of C-type virus. Dalton (1972) is of the opinion, however, that no true intracellular type-A particle is ever involved in C particle formation. In the present study, C particles were observed budding off the epidermal cell membrane. There is no evidence of intracisternal A particles being enveloped by the outer cell membrane, as in the relationship between intracytoplasmic A particles and intercellular B particles in mouse mammary tumours (Bernhard, 1958).

Since tumour production after SC treatment took longer than after G treatment at the dose levels used in this study, it would seem logical to suppose that the age of the mice might be more important than the difference in treatment. However, the age of the animals had no effect on A-particle formation. Tumours produced after relatively short-term treatment with polycyclic hydrocarbons possessed large numbers of A particles, as did tumours produced by longer-term treatments with SC and G. Since the A-type and C-type particles in this system are morphologically very similar, one cannot discount a relationship between the two. It may well be that BALB/c mice of greater age are less able to resist a transformation from inactive A particles to active C particles. However, this must remain purely speculative, as no direct relationship between the two has so far been demonstrated.

In conclusion it would seem that in BALB/c mouse epidermis the presence of visible C-type virus particles is not essential for tumour production. Their presence in skin tumours induced by SC could possibly be attributed to the age of the mice in this group at the termination of the study. The occurrence of C particles in this type of tissue is interesting in that they are usually associated with tumours of connective tissues and the haemopoietic and reticulo-endothelial system.

The work was supported by the Tobacco Research Council, London.

REFERENCES

BERNHARD, W. (1958) Electron Microscopy of Tumour Cells and Tumour Viruses. *Cancer Res.*, **18**, 491.

BERNHARD, W. (1960) The Detection and Study of Tumour Viruses with the Electron Microscope. *Cancer Res.*, **20**, 712.

BIBBY, M. C. & SMITH, G. M. (1975) Increase in Type A Virus Particles Induced in BALB/c Mouse Epidermis during Chemical Carcinogenesis. *Br. J. Cancer*, **32**, 660.

BROOKS, R. E. (1970) Lung Tumour Bearing Strain A Mice with Coincident Leukemia. An Electron Microscope Study. *Cancer Res.*, **30**, 1534.

BUCCIARELLI, E. (1972) Particelle Virali C e A Intracisternali Nei Tumori Pulmonari Spontanei di Topi BALB/c/Cb/Se. *Lav. Anat. Pat. Perugia*, **32**, 109.

BUCCIARELLI, E. & RIBACCHI, R. (1972) C-type Particles in Primary and Transplanted Lung Tumours Induced in BALB/c Mice by Hydrazine Sulfate; Electron Microscope and Immunodiffusion Studies. *J. natn. Cancer Inst.*, **49**, 673.

DALTON, A. J. (1972) RNA Tumour Viruses—Terminology and Ultrastructural Aspects of Virion Morphology and Replication. *J. natn. Cancer Inst.*, **52**, 483.

DALTON, A. J. & HAGUENAU, F. (1973) *Ultrastructure of Animal Viruses and Bacteriophages*—An Atlas. New York: Academic Press.

DALTON, A. J., POTTER, M. & MERWIN, R. M. (1961) Some Ultrastructural Characteristics of a Series of Primary and Transplanted Plasma-cell Tumours of the Mouse. *J. natn. Cancer Inst.*, **26**, 1221.

DAVIES, R. F. & DAY, T. D. (1969) A Study of the Comparative Carcinogenicity of Cigarette and Cigar Smoke Condensate on Mouse Skin. *Br. J. Cancer*, **23**, 363.

FRIEDLANDER, M. & MOORE, D. H. (1956) Occurrence of Bodies within Endoplasmic Reticulum of Ehrlich Ascites Tumor Cells. *Proc. Soc. exp. Biol.*, **92**, 828.

GROSS, L., FELDMAN, D., DREYFUSS, Y., EHRENREICH, T. & MOORE, L. A. (1976) C-type Virus Particles in Urethan-induced Pulmonary and Renal Carcinomas in Cell-graft Transmitted Carcinomas and Infiltrate-induced Lymphomas in Mice. *Cancer Res.*, **36**, 181.

GUILI, C. DE, HANAFUSA, H., KAWAI, S., DALES, S., CHEN, J. H. & HSU, K. C. (1975) Relationship between A-type and C-type Particles in Cells Infected by Rous Sarcoma Virus. *Proc. natn. Acad. Sci. U.S.A.*, **72**, 3706.

HOWATSON, A. F. & MCCULLOCH, E. A. (1958) Virus-like Bodies in a Transplantable Mouse Plasma Cell Tumour. *Nature, Lond.*, **181**, 1213.

KAKEFUDA, T., ROBERTS, E. & SUNTZEFF, V. (1970) Electron Microscopic Study of Methylcholanthrene-induced Epidermal Carcinogenesis in Mice. Mitochondrial Dense Bodies and Intracisternal A-particles. *Cancer Res.*, **30**, 1011.

MYERS, D. D., MEIER, H. & HUEBNER, R. J. (1970) Prevalence of Murine C-type RNA Virus Group Specific Antigen in Inbred Strains of Mice. *Life Sci.*, **9**, 1071.

PARSONS, D. F., DARDEN, E. B., LINDSLEY, D. L. & PRATT, G. T. (1961) Electron Microscopy of Plasma-cell Tumours of the Mouse. *J. biophys. biochem. Cytol.*, **9**, 353.

SMITH, G. H., ANDERVONT, H. B. & DUNN, T. B. (1970) Attempts to Detect Nodule-inducing Virus in Strain RIII. *J. natn. Cancer Inst.*, **44**, 657.

SUGGESTIONS for the classification of oncogenic RNA viruses (1966) *J. natn. Cancer Inst.*, **37**, 395.

TARIN, D. (1967) Sequential Electron Microscopical Study of Experimental Mouse Skin Carcinogenesis. *Int. J. Cancer*, **2**, 195.

WHITEHEAD, J. K. & ROTHWELL, K. (1969) The Mouse Skin Carcinogenesis of Cigarette Smoke Condensate Fractionated by Solvent Partition. *Br. J. Cancer*, **23**, 840.

WIVEL, N. A. & SMITH, G. H. (1971) Distribution of Intracisternal A-particles in a Variety of Normal and Neoplastic Mouse Tissues. *Int. J. Cancer*, **7**, 167.

YASAZUMI, G. & HIGASHIZAWA, S. (1956) Electron Microscope Study of Sections of Ehrlich Mouse Ascites Tumours. *Gann*, **47**, 527.